# First Overview of Zoological Medicine on Iberian Countries

**DOI:** 10.3390/vetsci11100512

**Published:** 2024-10-17

**Authors:** Fábio Cardoso-Freitas, Vanessa Silva, Albert Martinez-Silvestre, Ângela Martins, Patrícia Poeta

**Affiliations:** 1 University of Trás-os-Montes and Alto Douro (UTAD), 5000-801 Vila Real, Portugal; al53178@alunos.utad.pt (F.C.-F.); angela@utad.pt (Â.M.); 2 MicroART—Microbiology and Antibiotic Resistance Team, Department of Veterinary Sciences, University of Trás-os-Montes and Alto Douro (UTAD), 5000-801 Vila Real, Portugal; 3 Associate Laboratory for Green Chemistry (LAQV-REQUIMTE), University NOVA of Lisboa, 1099-085 Lisboa, Portugal; 4 Department of Genetics and Biotechnology, University of Trás-os-Montes and Alto Douro (UTAD), 5000-801 Vila Real, Portugal; 5 Functional Genomics and Proteomics Unit, University of Trás-os-Montes and Alto Douro (UTAD), 5000-801 Vila Real, Portugal; 6 Catalonian Reptiles and Amphibians Rescue Centre (CRARC), 08783 Barcelona, Spain; 7 Veterinary and Animal Research Centre (CECAV), University of Trás-os-Montes and Alto Douro (UTAD), 5000-801 Vila Real, Portugal; 8 Associate Laboratory for Animal and Veterinary Sciences (AL4AnimalS), 5000-801 Vila Real, Portugal

**Keywords:** exotic pets, exotic animals, wildlife, zoo, Veterinary Medicine, Portugal, Spain

## Abstract

The role of veterinary doctors, particularly those dedicated to Zoological Medicine, is becoming increasingly relevant and essential within the One Health framework. Educational and employment opportunities in this field vary widely across Europe, prompting us to characterise the sector in Portugal and Spain. A survey was conducted, resulting in 169 valid responses from veterinarians. Respondents were characterised based on gender, age, work environment, the highest level of education, additional training, whether they attended zoological medicine classes at university and their perception of the adequacy of that training. Further factors included years of experience, the percentage of their work involving zoological medicine, sectors they work in, the types of animals they treat, their confidence in treating these animals, sources of information they use, and membership in relevant professional associations. Relationships between these variables were examined. The results describe Iberian professionals working with non-traditional pets, wildlife, and zoo animals. Notably, only half of these professionals had taken zoological medicine classes at university; among those, the majority felt they inadequately prepared them for their work. Given the findings, enhancing the training and resources available to these professionals is crucial to prepare them for their roles better.

## Simple Summary

Understanding the path to becoming a veterinarian who works with exotic and wild animals may not be easy, so using a questionnaire, we aimed to describe and characterise these professionals. We found that most veterinarians felt their undergraduate formation was inadequate in preparing them for their work. Thus, the results showed where the needed changes would be made so that these professionals feel well-prepared for their work.

## 1. Introduction

### 1.1. Zoological Medicine

The term “exotic animal medicine” is often used interchangeably with the preferred term “Zoological Medicine” [1,2]. Zoological Medicine (ZM) can be defined as “the Veterinary Medicine of pets, wild or zoo species, excluding traditional domestic animals, and integrating the principles of ecology and conservation, applied to both natural and artificial environments” [3].

ZM is perceived as a recent development compared to other core subjects of Veterinary Medicine. However, its roots can date back to 1831 with the first zoo veterinarian [4]. The American Board of Veterinary Specialists (AVMA) recognised ZM as a speciality in 1988 [4], and the European College of Zoological Medicine (ECZM) followed in 1999 [5]. Today the ECZM recognises five specialties: Avian, Herpetology, Small Mammal, Wildlife Population Health, and Zoo Health Management [5].

Opportunities to enrol in ZM education vary widely across Europe, partly due to the country’s diversity [6]. In some cases, ZM has only recently been fully incorporated into formal veterinary training [3,7]. Subsequently, additional postgraduate formation is necessary to become a ZM veterinarian [3,8,9,10]. In response to the barriers identified by the European Board of Veterinary Specialisation, a new path to veterinary specialisation was announced to make it more accessible and flexible [11].

### 1.2. A One Health Perspective on Zoological Medicine

One-Health views the health of all species as interconnected and balanced [12], leading to increased awareness of ZM’s relevance [3].

The latest report on human zoonoses indicated an increase in notification rates for most zoonoses, although those causing the highest number of cases remained stable compared to the 2022 report [13].

In the post-COVID-19 era, veterinarians are seen as crucial sources of information regarding zoonoses, reinforcing their key role in education and prevention [12,14]. Even before COVID-19, the importance of education and prevention in ZM was highlighted to protect humans against zoonotic transmission [15]. ZM veterinarians can consult taxa or species-specific recommendations to safeguard human and public health [16,17,18,19,20,21,22,23].

ZM practice can be divided into three main application sectors: zoological parks (Zoos), wildlife rehabilitation centres (WRCs), and non-traditional companion animals (NTCAs), mainly in veterinary clinics and hospitals [3].

Each sector also has specific recommendations for facing zoonotic diseases [24,25,26,27,28]. NTCAs are increasingly popular as pets [3], posing a higher risk for zoonotic infections due to certain human-companion animal behaviours [7,29]. In zoos, a recently published handbook includes a specific chapter on disease surveillance planning covering zoonoses [30]. These organisations have evolved from exhibition places to institutions vital to welfare, conservation, education, and research [3]. Over 60% of zoonoses originate from animals, often linked to wildlife [12]. Thus, preserving nature and ecosystem services is crucial, alongside understanding activities that lead to zoonotic disease emergence [31]. WRCs act as sentinel centres, providing medical care to wild animals while monitoring zoonotic diseases [3].

### 1.3. Evidence-Based Practice in Zoological Medicine

Evidence-based practice in Veterinary Medicine is lacking. It involves using clinical expertise, client values, preferences, and the most relevant research evidence for clinical decisions [32]. Even in widely used ZM formularies and textbooks, work must still be done to meet this standard [33,34].

Reporting primary research results is critical in knowledge creation [35]. However, ZM professionals may find this information scattered across various journals [36]. Attending conferences is vital for accessing timely or otherwise unavailable information [37,38]. Although information retrieval and dissemination evolved towards open access, it requires awareness of the limitations and dangers associated with technology misuse [39].

### 1.4. Decision-Making Process for Zoological Medicine Treatments

Due to anatomical and physiological variations, drug delivery in ZM faces challenges, requiring species-specific techniques to avoid iatrogenic complications [40]. There is a significant need for pharmacokinetic and pharmacodynamic studies in ZM since each drug compels specialised considerations for different species [41]. Often, medications are prescribed without knowing their safety in a particular species, even if data exists for closely related species [42]. The lack of approved products for ZM species exacerbates the need to prepare and use drugs in a different way from what is indicated on the label (extra-label), which carries inherent risks [43].

Even with specific drug data, ZM clinicians may need to consider group treatments, weighing the pros and cons [44]. Multiple drug usage (polypharmacy) must also be carefully managed to assess drug interactions and their effects on the animal [45,46].

### 1.5. Zoological Medicine in Portugal and Spain

At the time of the writing, there was one ECZM specialist in Portugal (in wildlife population health) and 12 in Spain (5 in wildlife population health, 3 in small mammals, 2 in herpetology and 2 in zoo health management) [47]. The Spanish Small Animal Veterinary Association (AVEPA) has certified 27 NTCA’s veterinary specialists through various Spanish universities [48]. In the first European ZM study, 12% of the respondents worked specifically with zoo or exotic animals [7]. In the latest Vetsurvey, 16% worked with NTCA’s and 3% worked with Zoos [49]. Data from the last survey on European veterinary professionals estimated that 20% of Portuguese and 16% of Spanish veterinarians work with NTCA’s. However, when asked by the authors, the average workload of Portuguese veterinaries was 1.5% for NTCAs and 0.5% for Zoos, while for Spanish veterinarians, it was 2% and 0.3%, respectively [49].

Eurostat estimates that there are about 7,148,000 exotic animals in Portugal and Spain combined [49]. The pet food industry estimated 981.000 NTCAs in Portugal in 2022 (641,000 ornamental birds, 83,000 aquaria, 218,000 small mammals, 39,000 terraria) and 10,669,000 NTCAs in Spain (6,991,000 ornamental birds, 691,000 aquaria, 1,523,000 small mammals, 1,464,000 terraria) [50].

This manuscript aims to investigate ZM professionals in Portugal and Spain to understand them better and align with European and global goals.

## 2. Material and Methods

For this study, we conducted a scientific review to understand the scope of this professional framework and developed a survey using Google Forms to gather veterinarians’ opinions.

We created a database of potential contacts from available websites in Portugal and Spain, resulting in 10 associations/organisations, 13 ECZM diplomates, 89 Zoos, 90 WRCs, 155 private veterinary clinics, hospitals, and other services for NTCAs, and one Facebook group.

The survey was distributed via email, social media, and colleague-to-colleague networks using a short link, an image with a brief description, and a QR code. Participation rights were ensured, including voluntary participation, anonymity, informed consent, and data confidentially, following applicable regulations (Declaration of Helsinki, Oviedo Convention, General Data Protection Regulation and the European Code of Conduct for Research Integrity ESF/ALLEA).

Eligible participants were licensed veterinarians residing in Portugal or Spain and could complete the online survey from January 29th to April 24th, 2024. All statistical analyses and descriptions were performed using software Excel version 16.86 and JMP^®^ version 17.2.0. The Chi-square (X^2^) or Fisher’s Exact tests were used where appropriate, and statistical significance was considered when *p* < 0.05.

Based on the percentages discussed in previous European surveys [7,49], we estimated a population of 6523 veterinarians. We set a confidence interval of 95% and an estimated true proportion of 0.08 (the average estimated proportion from both surveys and countries). Using the EPITOOLS epidemiological calculator, we anticipated a minimum sample size of 113 responses.

## 3. Results

There were 173 respondents; however, four were excluded: 1 for being from the United Kingdom and 3 for not being licensed veterinarians. Therefore, the responses of 169 respondents were included in this study.

**Figure 1:**
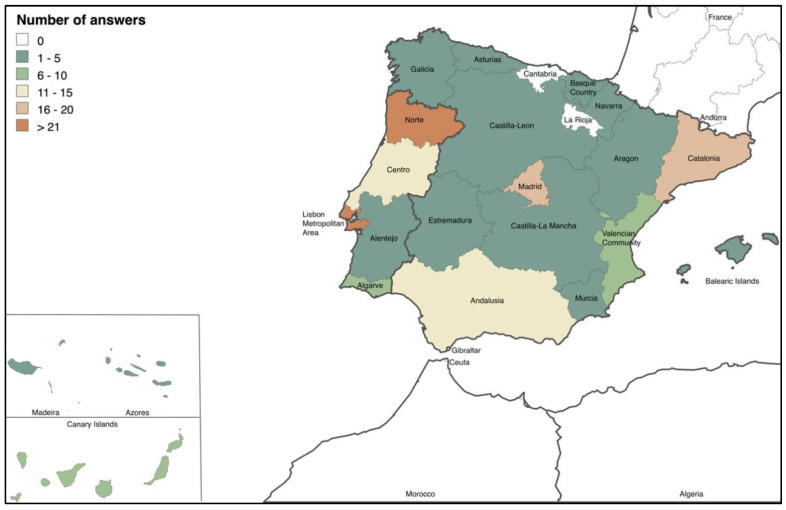
Distribution by number of answers from veterinarians (according to NUTS II).

When asked about their location, 52% were from Spain (SP), 48% from Portugal (PT), 75% from urban areas, and 25% from rural regions. The distribution of answers is reported in Figure 1.

The respondents were 68% female and 32% male, with varied representation of ages: 29 or younger (28.4%), 30–39 years (29.6%), 40–49 years (25.4%), 50–59 years (13.0%), and 60 or older (3.6%). There was a significant age difference between the two countries (*p* = 0.034).

Regarding education attainment, there was a highly significant difference (*p* < 0.0001) in the highest degree obtained between PT and SP, detailed further in Table 1.

To evaluate experience, respondents were categorised into ranges of years of experience, resulting in no significant difference between countries: <5 years (34.3%), 6–10 years (20.1%), 11–15 years (12.4%), 16–20 years (13.0%), 21–25 years (10.1%), 26–30 years (4.1%), and over 31 years (5.9%).

**Figure 2:**
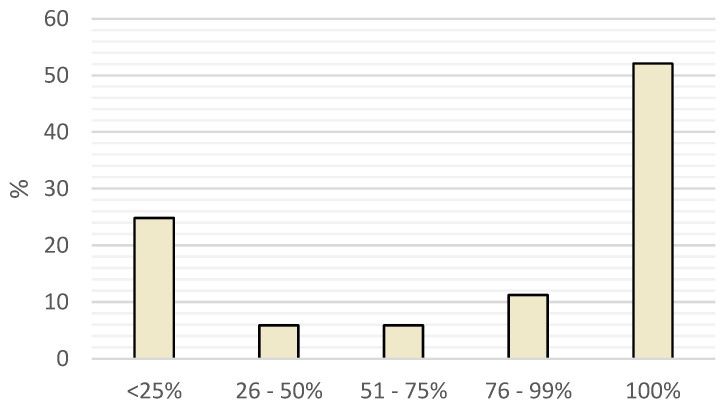
Zoological Medicine workload (x: percentage of ZM, y: proportion of the respondents).

There was no significant difference between countries when explicitly discussing ZM workload (Figure 2). Still, there is a twofold higher odd of having more than 50% ZM workload if you have any post-graduate degree other than the country’s standard DVM or MSc for being a licenced veterinarian ([1.1|4.8] *p* = 0.025).

Concerning undergraduate education, 50.9% of the testifiers didn’t have ZM classes, with no significant difference between PT and SP. However, only 12.4% of the ones who did, considered that prepared them for their work, with a very significant difference amongst countries ([1.9|23.7] *p* = 0.001), with the probability to evaluate the classes as suited to prepare for work being 6.7 times higher in SP than PT (32% vs. 7%). Also, compared with the experience, there was a highly significant difference between groups (*p* < 0.0001), showing a growing tendency in the prevalence of ZM classes from the most experienced (with fewer ZM classes) to the least experienced (with more ZM classes).

Reflecting on other education, with no specific degree but more in line with continuous education, results stated that 72.2% had an externship, 87.6% assisted webinars and 67.5% attended weekend formations/congresses (with no significant difference amid nations). In comparison, 45.0% joined intensive courses, but there was a significant difference between PT and SP ([1.1|3.8] *p* = 0.030), with two times higher odds of attending these courses from Spanish practitioners (PT 36% vs. SP 53%).

When asked, 43.3% of the respondents were members of an international ZM-related association (with no significant difference between both countries), and 33.1% were members of a national ZM-related association, with Spanish practitioners being nine times more likely to be a member ([4.1|20.6] *p* < 0.0001).

**Figure 3:**
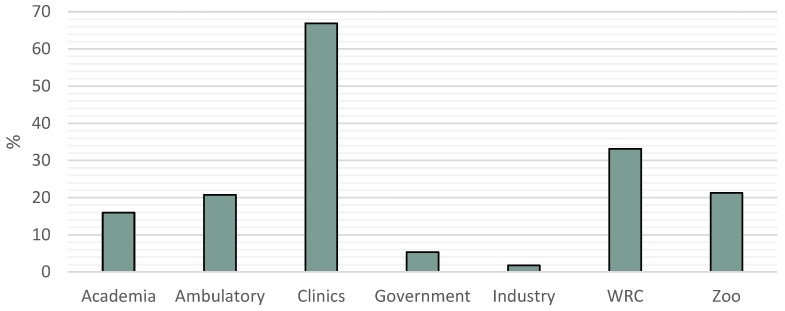
Sectors of Zoological Medicine.

Bearing in mind the possible different sectors involving ZM (Figure 3), the results showed no significant difference between countries except for ambulatory services, where there was a highly significant difference (*p* < 0.0001), with veterinarians from Portugal having a greater probability of doing ambulatory (PT 35% vs. SP 8%). Also, 43.8% of the professionals worked in more than one sector, with no significant difference between countries.

**Figure 4:**
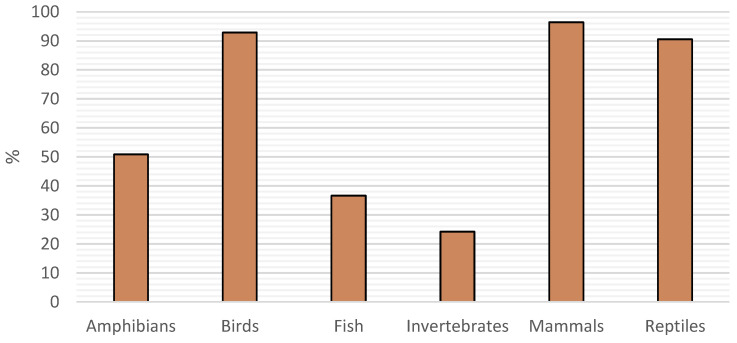
Prevalence of attendance of various animal groups.

Looking over the responses, we managed to get an idea of the prevalence of attendance of different animal groups (Figure 4). Overall, there was no significant difference between the two countries other than in reptiles, where there were a 5.42 times greater odds of seeing reptiles in SP than in PT ([1.5|19.8] *p* = 0.007).

**Figure 5:**
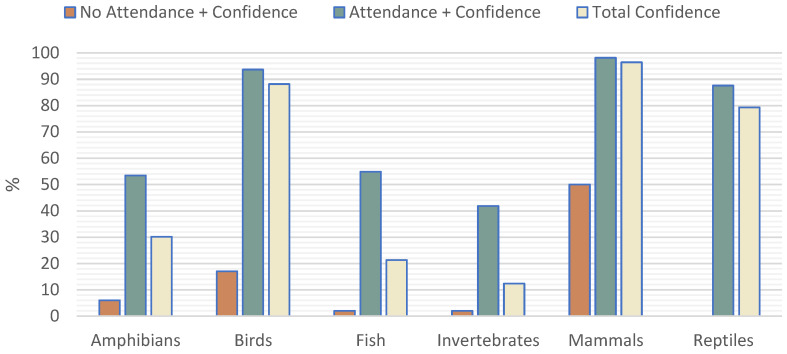
Comparison between total confidence in treating groups of animals and the difference in those attending or not attending those same animals.

Once again, there was no significant difference between the two countries regarding confidence in treating those groups of animals (Figure 5).

Scrutinising the variables more and evaluating the dependence with confidence in treating those animals, we came up with some outcomes outlined in Table 2 (should be interpreted as the probability of feeling confident in treating those animals when answered positively to the variable on the left column).

When working in ZM more than 50% of the time, it shows that clinicians feel three to eight times more likely to feel confident when treating amphibians, birds and reptiles. When working in ZM 100% of the time, clinicians were two to five times more likely to feel confident treating those same animals. Meanwhile, in the other animal groups, the workload in ZM does not seem significant for feeling confident in treating them. On the other hand, veterinarians with a postgraduate degree had a double to triple greater odd of feeling confident in treating amphibians, fish and reptiles.

Another fact is that there is a highly significant difference for veterinarians seeing a particular group of animals if they feel confident in treating those same groups rather than the ones who don’t (Table 3).

**Figure 6:**
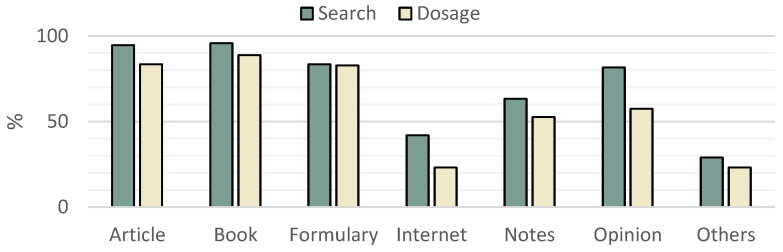
Percentages of usage of different sources to search for drugs and dosages.

When asked about the main sources where veterinarians searched for drugs and search for dosages, the compilation is summarised in Figure 6. There was a very significant difference among veterinarians from SP who were twice more likely to use the internet for searching drugs (SP 52% vs. PT 31%; [1.3|4.6] *p* = 0.005), to use notes for searching dosages (SP 67% vs. PT 59%; [1.2|4.3] *p*= 0.009) and to ask for colleagues’ opinions for dosages than veterinarians from PT (SP 84% vs. PT 79%; [1.2|4.3] *p* = 0.013).

Dissecting the characteristics of the sources used by these professionals, it was predictable that 95.1% consulted sources in English, without significant differences between PT and SP. Other than English, 61.0% consulted Spanish sources, 36.7% Portuguese and 16.6% other languages. Spanish veterinarians were twice more likely to consult Spanish sources ([1.1|3.9] *p* = 0.027), and Portuguese veterinarians’ probability of using Portuguese or sources in other languages was greater than Spanish practitioners (*p* < 0.0001; *p* = 0.002; respectively).

Still, about sources, 78.7% of the veterinarians surveyed verify their sources, and 81.1% of them think sources are scarce, with Spanish veterinarians having a 4.2 times higher probability of feeling it (SP 91% vs. PT 70%; [1.8|10.0] *p* = 0.001). A more delicate theme is the trust relied on the sources; though 87.0% do trust the sources, there’s an 8.7 times greater chance of trusting by SP ([2.5|30.6] *p* < 0.0001), a 4.8 times greater chance of trusting by professionals with a post-graduate degree ([1.3|16.8] *p* = 0.009) and a four times greater chance of trusting by veterinarians who are members in international ZM-related associations ([1.3|12.3] *p* = 0.012).

Regarding generating new sources, 63.9% of the repliers participated in some form of publication and 30.8% participated in an article in the last 24 months (with 48.2% participating in some form of publication and participating in an article in the previous 24 months). The results of inspecting the dependence of relevant variables and both types of generation of new sources are described in Table 4.

Veterinarians who belong to a national or international ZM association, have a workload of over 50%, or are full-time ZMs have a doubled to tripled probability of having participated in a publication. Colleagues with a postgraduate degree, ECZM degree, or membership in a ZM association are two to six times more likely to have collaborated on an article in the last two years.

## 4. Discussion

The data collected regarding gender and age aligns with the latest study of the profession in both countries [49].

Notably, most newly graduated veterinarians in SP typically achieve a DVM equivalent, whereas Portuguese practitioners generally attain a Master’s equivalent following the Bologna Process. Additionally, SP is seeing an increase in accreditation for ZM specialists, unlike PT, where no such accreditations exist [48]. Furthermore, there are two approved residency training programs for ECZM in SP, while none exists in PT [5]. This information helps explain the differences between the two countries regarding educational qualifications such as DVM, MSc, Specialist ZM and ECZM.

When comparing the years of experience with data from practitioners across Europe, our sample appears to be less experienced than expected [49].

Considering the literature supporting the need for additional postgraduate formation to work with ZM [3,8,9,10], the information gathered here corroborates the idea of a market demanding more specialised expertise [7], where professionals who do have additional degrees end up having more ZM workload than their counterparties. This fact may be closely related to the feeling of unpreparedness from undergraduate ZM classes seen here, but already identified [3,10], and the difference between countries, also stated before [6]. However, the statistics also show the growing effort to tackle this feeling as ZM classes and formation become more prevalent, as it was hypothesised by several authors [1,3,6,7,8,10].

The effort and interest in enrolling in continuous education programs align with what is asked of these professionals [6,9].

It was expected that national association membership would be significantly different because only Spanish practitioners have a ZM-related association [48]. However, Portuguese practitioners can join this and other associations, whether Iberian or international.

When we examined the sectors in which these professionals work, some details are highlighted when compared to the country’s employment fields [49]: private practice is in line with the data and is as predicted the most common sector; academia is higher; government and industry are lower; ambulatory practice, WRC and Zoo we don’t have data to compare to; the percentage of veterinarians who dedicate their time to more than one sector is higher than the data. All these may be justified by the small number of professionals and the fact that it is still a growing professional field.

To get a more general idea of the type of animals seen by veterinarians, we chose to evaluate the prevalence of bigger groups of animals rather than the frequency of consults with particular exotic pet species [7,10]. However, we get the same general idea that mammals, birds and reptiles are the most seen.

The information presented here agrees with the literature, which says that those seeing these animals feel more confident [10]. It is, in fact, one of the major variables affecting confidence in all groups, while workload and having a postgraduate education and degree influence certain specific groups of animals (amphibians, birds, and reptiles).

Relatively, the data on the usage of different sources agrees with the literature on the lack of literature [32,34,39], and there is a feeling of trust in said literature with the given particularities [51]. Nevertheless, we’ve got different results from other related literature, like an increased percentage of respondents checking their sources and not such a big preference for formularies or notes as predicted [33]. Another interesting fact was that Portuguese veterinarians were more likely to use languages other than Portuguese or English, which can be explained by the versatility of Portugueses with foreign languages [52].

Reflecting on the publication rate in ZM, the data presented here shows an increased percentage of having published an article in the last 24 months than was expected [38]. Being a member of a ZM association, having a postgraduate degree, and being an ECZM diplomate are associated with higher probabilities of publishing an article. This suggests that these professionals may have more inclination or motivation to pursue publications [38].

## 5. Conclusions

More than ever, the role of veterinary doctors (not only veterinarians) is becoming increasingly relevant and necessary within the One Health concept. In this article, we emphasise professionals devoted to zoological medicine. We examine two neighbouring countries that may be on different paths on the same journey in advancing zoological medicine. Yet, they share a collective responsibility commonality in people, animals, culture, and wildlife. The detailed data helps us achieve our goal of characterising and better understanding these veterinarians.

Given the variables studied and the different sectors and animals impacted, we must enhance our efforts in preparing these professionals for their future roles by providing them with the appropriate tools to feel more confident in their work. This will lead to the production of more knowledge and, consequently, better care for both animals and people. Although improvements were made to meet current demands, the results indicate that different, optimised, and sector-specific approaches should be implemented at the undergraduate level. Furthermore, postgraduate education should be made more accessible to these professionals until these changes can be fully realised.

## Figures and Tables

**Table 1:** Highest degree of education obtained and difference between countries.

Degree	Total N (%)	Country by (*p*-Value)	Portugal N (%)	Spain N (%)
DVM	59 (34.9)	*p* = 0.008	20 (24.7)	39 (44.3)
MSc	58 (34.3)	*p* < 0.0001	47 (58.0)	11 (12.5)
Pg ZM	28 (16.6)	*p* = 0.862	13 (16.1)	15 (17.1)
PhD	7 (4.1)	*p* = 0.069	1 (1.2)	6 (6.8)
Specialist ZM	10 (5.9)	*p* = 0.002	0	10 (11.4)
ECZM	7 (4.1)	*p* = 0.010	0	7 (7.9)

Degree in Veterinary Medicine (DVM), Master’s (MSc), Postgraduate in ZM (Pg ZM), Doctorate (PhD), Specialization in ZM (Specialist ZM) and Diplomate of the European College of Zoological Medicine (ECZM).

**Table 2:** Cross-action between variables and confidence in treating these groups of animals.

Confidence	Amphib.	Birds	Fish	Inverteb.	Mammal	Reptiles
<25% ZM workload	**	***	NS	NS	*	***
>50% ZM workload	3.1 OR [1.3|7.3] **	8.5 OR [1.1|4.3] ***	NS	NS	NS	4.0 OR [1.8|8.9] ***
100% ZM workload	2.2 OR [1.1|4.3] *	5.0 OR [1.6|15.8] **	NS	*	NS	3.4 OR [1.5|7.6] **
postgraduate degree	2.3 OR [1.2|4.5] *	NS	3.2 OR [1.5|6.8] **	NS	NS	3.1 OR [1.3|7.7] *

NS—nonsignificant; OR—odds ratio; *—significant (*p* < 0.05); **—very significant (*p* < 0.01); ***—highly significant (*p* < 0.001).

**Table 3:** Cross-action between confidence in treating these animals and attending them.

Attendance	Amphib.	Birds	Fish	Inverteb.	Mammal	Reptiles
Confidence	17.9 OR [6.6|48.7] ***	73.5 OR [14.1|382] ***	63.8 OR [14.4|282] ***	32.6 OR [8.9|120] ***	53.3 OR [7.5|381] ***	***

OR—odds ratio; ***—highly significant (*p* < 0.001).

**Table 4:** Cross-action between variables and participation in publications and publication of an article in the last 24 months.

Participation in	Publication	Article in Last 24 Months
PhD	*	NS
Specialist ZM	NS	*
ECZM	NS	6.1 OR [1.1|32.6] *
Postgraduate degree	NS	2.4 OR [1.2|4.7] *
Nat. Association	2.5 OR [1.2|5.0] *	2.9 OR [1.4|5.7] **
Internat. Association	3.3 OR [1.7|6.5] ***	3.3 OR [1.7|6.6] ***
>50% ZM workload	2.7 OR [1.4|5.4] *	NS
100% ZM workload	2.4 OR [1.3|4.5] *	NS

NS—nonsignificant; OR—odds ratio; *—significant (*p* < 0.05); **—very significant (*p* < 0.01); ***—highly significant (*p* < 0.001).

## Data Availability

The datasets generated and/or analysed during the current study are available from the corresponding author upon reasonable request.
